# Development of a Pathway for Multidisciplinary Neurodevelopmental Assessment and Diagnosis in Children and Young People

**DOI:** 10.3390/children8111033

**Published:** 2021-11-11

**Authors:** Marion Rutherford, Donald Maciver, Lorna Johnston, Susan Prior, Kirsty Forsyth

**Affiliations:** 1 School of Health Sciences, Queen Margaret University, Edinburgh EH21 6UU, UK; mrutherford@qmu.ac.uk (M.R.); LJohnston2@qmu.ac.uk (L.J.); sprior@qmu.ac.uk (S.P.); kforsyth@qmu.ac.uk (K.F.); 2 Additional Support for Learning Service, Communities and Families, City of Edinburgh Council, Edinburgh EH8 8BG, UK

**Keywords:** guideline, autism, neurodevelopmental, pathway, assessment, diagnosis

## Abstract

There is a variable standard of access to quality neurodevelopmental assessment and diagnosis. People may have negative experiences, encountering lengthy waiting times, and inconsistent practices. Practitioners need guidance on standards and practices for assessment and diagnosis matched to new ways of working. In this paper, we present a new pathway and recommendations for multidisciplinary neurodevelopmental assessment and diagnosis for children and young people (<19 years), developed by the Scottish Government funded National Autism Implementation Team (NAIT). Our research used the Medical Research Council guidance for the development of complex interventions and included several iterative stages. Stage 1: *n* = 44 stakeholders attended an event on developing new practices for diagnosis and assessment. Stage 2: a literature synthesis was completed by the research team of clinical guidelines and diagnosis and assessment tools. Stage 3: an event with *n* = 127 stakeholders included discussion and debate of the data from stages 1 and 2. Recommendations and a draft pathway were written. Stage 4: successive drafts of recommendations and the pathway documentation were circulated among an advisory group, including multidisciplinary clinical experts and people with lived experience, until the final pathway was agreed upon. The finalised pathway includes guidance on terminology, assessment, diagnosis, triage, time standards and engagement of people with lived experience. The new pathway has been adopted by the Scottish Government. The pathway and associated documentation are freely available online for use by others.

## 1. Introduction

People with neurodevelopmental differences present with variations in one or more neurocognitive functions towards the extreme or otherwise out of the ‘normal’ range [1,2]. Common diagnoses include autism, developmental coordination disorder (DCD), developmental language disorder (DLD), intellectual disability (ID), foetal alcohol spectrum disorder (FASD), and attention deficit hyperactivity disorder (ADHD) (American Psychological Association (APA), 2013). These categories frequently overlap, and most individuals present with interrelated needs and requirements for support [3].

Accessing timely assessment and diagnosis is a known area of difficulty for people with neurodevelopmental differences, and there are few guidelines available to support practice. Delayed or missed diagnosis reduces access to help, and has a negative impact on wellbeing, participation and quality of life [4,5,6,7,8]. An overly deficit-focused approach is also unhelpful. Diagnostic assessment should include an understanding of the individual in context, and their strengths and goals [9]. An approach which considers ‘difference not deficit’ is important [10].

There has been a tradition of single condition guidelines and pathways [6,11,12]. The focus has been on the presence or absence of a single diagnosis, leading to communities of practice with knowledge, skill and capacity in relation to these areas (e.g., autism). As a consequence, services have become overspecialised, drastically underestimating the true degree of overlap across neurodevelopmental differences [13]. A singular focus on one presenting issue leads to the potential omission of other diagnoses (and supports). People may experience repetitive and lengthy assessment, a process which individuals and families find distressing and burdensome [4,14].

Movement from single condition pathways toward neurodevelopmental pathways is desirable. Although there are strong advocates for this, for example the ‘ESSENCE’ framework [3], bridging the gap into practice remains challenging [15]. There was no neurodevelopmental guideline for assessment and diagnosis in Scotland. In this research, commissioned by the Scottish Government, we present our work to address this gap.

### 1.1. Objective

With this paper, we present recommendations to guide multidisciplinary practice from pre-referral to diagnosis for children and young people aged 0–18 years. We focus on the 0–18 age range as we wish to develop future materials for post-18-year-old individuals, which will have a greater focus on employment, while this current guidance includes a greater focus on childhood years, covering the periods of primary and secondary education. In this research we have built on the United Kingdom Medical Research Council (MRC) guidance on the development of complex interventions [16] and engaged with stakeholders, professionals and people with lived experience. Recommendations were primarily derived from consultation in Scotland; however, they may be transferability to other countries with similar characteristics or issues.

### 1.2. The Scottish Context

In Scotland, services report increasing numbers of children and young people meeting the criteria for one or more neurodevelopmental diagnosis. The current prevalence of autism is 1.035% in Scotland [17]. The estimated prevalence of neurodevelopmental disorders (internationally) is around 10% [3]. Other Scottish neurodevelopmental prevalence data is not known; however, an analysis of 2019 school census data found that 15% of primary aged children and 20% of secondary aged children had an additional support need indicative of neurodevelopmental needs, including learning disabilities, learning difficulties, language or communication disorders, autism, social, emotional and behavioural difficulties, and mental health problems [18]. In Scotland, there are defined pathways for autism [19,20] and largely separate pathways for ADHD and other neurodevelopmental differences. Prior to the COVID-19 pandemic, services had begun implementing neurodevelopmental pathways, and others were considering this shift, but little synthesised guidance existed to support this important change in practice.

## 2. Methods

### 2.1. Team

This research was completed by the National Autism Implementation Team (NAIT). This team forms part of the 10-year Scottish Government autism strategy [21,22]. NAIT have a leadership role in this strategy, and have a remit to improve autism and neurodevelopmental diagnostic pathways across the lifespan through integration with education (in children) and with the employment sector in adults. The team includes clinical expertise in autism, occupational therapy, speech and language therapy, education, psychiatry, as well as researchers with expertise in complex interventions development and health systems research. The team has completed previous research on autism pathways, diagnosis, wait times, interventions and guidelines [6,7,19,20,23,24,25,26].

### 2.2. Medical Research Council Complex Interventions Development Framework

Intervention development methods were modernised through the publication of the MRC complex interventions development framework [16]. The MRC framework provided a systematic and cyclical approach to the development of new interventions [16]. Whilst quantitative statistical techniques, including the controlled trial or statistical meta-analysis, are important, such methodologies may not always be feasible. Qualitative methods are an important alternative [27,28]. A key aspect of such approaches is stakeholder involvement, which can be used to understand problems, identify priorities, and collaborate to develop solutions [29,30]. The MRC guidance provides a robust standard for intervention development and includes four phases: development, feasibility/piloting, evaluation and implementation [16]. The development phase, as recommended by the MRC framework, focusses on building partnerships, specifying the population, completing a literature review, collaborating with stakeholders, and developing and consulting on materials to support implementation [30].

In following the development phase of the MRC framework, an iterative multi-stage process was undertaken 2019–2021. This began with stakeholder engagement, followed by a literature review, further stakeholder engagement, and recruitment of an advisory group. We attempted to find and synthesise data focused on the research questions as listed below:

1. How should people with neurodevelopmental differences be identified for diagnostic assessment?
2. Which documents and guidelines are relevant to implementing a neurodevelopmental pathway?
3. Which assessment tools are applicable ‘pre-referral’ to identify a need for neurodevelopmental assessment, relevant to all diagnoses?
4. What are the key considerations for assessment and triage?
5. Which ‘disorder specific’ assessment tools should be considered?
6. What are the key considerations for making a diagnosis, and reporting this to individuals and their families?
7. What time standards should be applied to a neurodevelopmental pathway?
8. How can we involve lived experience in the development of pathways?

### 2.3. Literature Review Methods and Analysis

The research team completed a rapid review of clinical guidelines and available diagnosis and assessment tools. We began searches in December 2019 of Cochrane, Medline, CINAHL, PubMed and Google for pre-existing clinical guidelines and diagnosis and assessment tools for neurodevelopmental diagnoses: autism, developmental coordination disorder (DCD), developmental language disorder (DLD), intellectual disability (ID), foetal alcohol spectrum disorder (FASD) and attention deficit hyperactivity disorder (ADHD). Search terms included ICD-10 and DSM-5 definitions, acronyms and other terminology alternatives for each neurodevelopmental diagnosis, as well as terms for clinical guidelines. The list of assessments for inclusion was developed by consultation with clinicians about local, national and international practice, and consultation with experts, as well as the reviewing of papers to identify commonly used tools in research. Based on these criteria, we identified assessments most commonly studied, most used in research and most used in practice. The focus was therefore on widely used and studied measures, rather than on providing a systematic overview. We revisited the literature throughout the development of the pathway. We set up automatic alerts to monitor the relevant literature for updates.

Summary tables were developed using content analysis and narrative summary methods [31]. Recommendations and guidance were synthesised across the available clinical guidance/guidelines in relation to the key questions of the study. Summaries of assessment tools were prepared, with overview of each tool, users, population, and constructs. A matrix was constructed to indicate which tools were relevant to which neurodevelopmental diagnosis. Tables of diagnostic factors for clinicians were derived. The tables contained descriptions and likely indicators associated with the DSM-5 criteria for autism, ADHD, ID, DLD, DCD and FASD. A matrix was developed to display the complex set of observations and information required in relation to diagnostic criteria. The information developed was used to facilitate stakeholder discussion, and eventually formed a key part of the pathway documentation.

### 2.4. Stakeholder Engagement Methods and Analysis

Stakeholders were identified for involvement through professional and user networks, and represented every health-board area of Scotland. Senior professionals and people with lived experience were included throughout. Stakeholders included people with lived experience, families and local autism advocacy groups. Professionals included psychiatrists, psychologists, teachers, occupational therapists, speech and language therapists, paediatricians, and academics. Stakeholders also included members who were involved in government and regulatory bodies.

We used group-work, workshops, discussion and debate with stakeholders, applying interactive and participatory methods [32,33] over a series of face-to-face events with follow up review, phone calls, emails, individual meetings, small group discussion and videoconferencing. The first group of stakeholders (*n* = 44) were invited to attend an initial event. Initial priorities and directions were identified. The key problem under discussion was improvement of diagnosis and assessment, having identified the service and personal costs of missed or delayed diagnosis. There were initial ideas about improvements which could be implemented, and the format or delivery of the proposed pathway. Knowledge about the scope and nature of the problem and possibilities for improvement were discussed, including personal experiences, the work of practitioners, policymakers, and researchers [29,30]. The research team-maintained notes of the discussions, synthesising key themes and main points as discussion documents, which were further shared with the group via email and videoconference. During this period, the review of evidence was undertaken, and draft pathway documentation produced. Next, a larger group of stakeholders (*n* = 127) attended a face-to-face event. This event included review of the draft pathway, review of the literature review outputs, and identification of themes and feedback. This event helped us to understand issues and generate new ideas concerning the pathway. Additionally, “buy-in” was required from the community, with understanding gained on the feasibility, acceptability and potential future engagement of people with the pathway. Each stage included the careful review of collected information and frequent return to primary documents and stakeholders as necessary. Summaries were derived and synthesised using content and thematic analysis [34]. As the analysis progressed, more refined descriptions and recommendations were created, and detailed prototype pathway documentation and content was written.

### 2.5. Advisory Group

An advisory group (*n* = 25) was recruited to consult on and finalise the pathway, representing an expert multi-disciplinary group comprising occupational therapy, clinical psychology, psychiatry, child and adolescent mental health services, paediatric medicine, general practice (family medicine), teaching, speech and language therapy, government representatives, and people with lived experience. Most individuals in the advisory group had participated in one or both of the previous stakeholder events. This was a coproduction team, with stakeholders participating extensively with researchers to finalise the content and documentation of the pathway. As a team, we regularly communicated to discuss and agree upon content. Debate and review were completed and supported by email and videoconferencing. Three cycles of detailed review of draft documentation were completed with the advisory group (December 2020, February 2021 and May 2021). As stakeholders had stressed the need for high quality documentation, a graphics design process was undertaken when the content had been finalized. The pathway was then made freely available online [35].

### 2.6. Ethics

All stakeholders were volunteers, and were free to withdraw at any time for any reason. Stakeholders were provided with information about the project and consented to participate in consultation events and activities. People with lived experience were volunteers who self-identified as having neurodevelopmental differences, they self-selected to participate, and were identified through pre-existing health and social care programmes for public and patient involvement and government engagement. This work was carried out in accordance with the relevant ethical standards of institutional and national practice in Scotland, and in-line with the Declaration of Helsinki.

## 3. Results

The final neurodevelopmental pathway was designed to cross professional boundaries and facilitate collaboration between medical professionals, mental health professionals, education professionals, allied health professionals, service users and families. Supplementary File S1 contains an overview of the full pathway. The content of the guidance is detailed more fully in the sections that follow.

### 3.1. How Should People with Neurodevelopmental Differences Be Identified for Diagnostic Assessment?

There was consensus that neurodevelopmental assessment and diagnosis should be supported by a multi-disciplinary team (MDT), with suitable training and mix of skills and professional groups. Clinical views, assessment and observations are mapped to ICD 11 or DSM 5 criteria. Most commonly, assessments are likely to result in more than one diagnosis, including autism, ID, DLD, ADHD, DCD and FASD. It was noted that classification systems focus on those who have a specific aetiology and/or extreme impairment. These perspectives were contrasted with a neurodiversity paradigm which rejects the idea of ‘normal’ neurocognition. There was strong debate, leading to a consensus that it was also helpful to consider that children may require a neurodevelopmental assessment when presenting with a need as below:

- Communication and social interaction,
- Emotional regulation,
- Co-ordination and/or movement,
- Developmental delay or difficulties across a range of skills,
- Intellectual development or a need for adaptation to support learning,
- Reduced independence in daily routines and activities.

It was noted that referrers, parents or other family members may see the situation differently and describe what they ‘see,’ and not necessarily in terms of specific diagnoses. The following were agreed upon as indicators in need of need of further assessment:

- Behaviour changes,
- Distress,
- Obsessions and compulsions,
- Changes in patterns of eating, sleeping, activity levels or passivity.

Rather than seeing an ‘in-person problem’ the position taken within the pathway (taking account of the views of people with lived experience), supports the right to access diagnostic assessment, even if the individual is relatively unaffected [36]. Whilst this may suggest that the right adjustments are already in place, it may also suggest that the individual is masking or camouflaging [37]. There is a need to balance between efficiency and comprehensiveness [38], and diagnostic assessment should also aim to create a detailed profile of strengths and concerns to inform planning. A lifespan perspective is therefore important. Neurodevelopmental differences are enduring, and understanding which anticipatory adaptations might be helpful at different stages is enhanced when diagnosis is clear.

### 3.2. Which Documents and Guidelines Are Relevant to Implementing a Neurodevelopmental Pathway?

There was no single Scottish neurodevelopmental guideline. Assessment may result in overlapping diagnoses, and Some, but not all, had a guideline. We identified and summarised the available documentation, and drew on these for the pathway (Table 1).

### 3.3. Which Assessment Tools Are Applicable ‘Pre-Referral’ to Identify a Need for Neurodevelopmental Assessment, Relevant to All Diagnoses?

Information gathered at the pre-referral stage supports timely and proportionate professional involvement [23]. Avoidance of duplication is desirable. Before commencing a neurodevelopmental assessment, we identified the following suggestions for professionals:

- Review available reports from professionals.
- Indicators for concern can be identified through screening, through the parent/carer or family, and from interviews and observations of the person in their typical environments (e.g., school, home, community).
- Gain the persons consent to include others as necessary, and communicate the likely process (e.g., through a ‘leaflet’).
- Develop a document containing the referral indicators; this could include the person’s current needs, wishes, and environments, as well as the views of the parent/carer or family.
- Gather key information on early development, including alcohol exposure and other relevant family history as required.

In line with the available clinical guidelines, we recommend that pre-referral information can be gathered through:

- Interview (remote or face to face),
- naturalistic observation in typical environments,
- standardised or non-standardised questionnaires or tools,
- contextual assessment approaches,
- locally-used approaches/tools (these are not diagnostic but provide qualitative information—local teams should agree on tools which are used for all individuals, and tools which are used in specific circumstances).

We reviewed and summarised key tools that are commonly used and recommended either in published clinical guidelines or by expert stakeholders, and were applicable to all diagnoses in the pre-referral stage (Table 2).

### 3.4. What Are the Key Considerations for Assessment and Triage?

Standards for formal neurodevelopmental assessment agreed with stakeholders are presented in Table 3. Recommendations focus on early identification of ‘core’ and ‘complex’ cases, presented in Table 4. This categorisation was proposed to support proportionate intervention and avoid duplication. In short, if a team are confident of a straightforward conclusion towards diagnosis (i.e., a ‘core’ case), there is no need for appointments with several sets of professionals. Where there is less complexity, multi-stage assessment may not be required, and assessments are completed locally by a smaller team. Diagnosis can be completed rapidly in most cases. If there is greater complexity, identification of such is completed promptly. More appointments may be necessary, and enhanced staff skill required, with time allocated for additional standardised diagnostic tools and formulation.

It is desirable, both for those referred and in order to deploy resources, that only those for whom it is relevant undergo diagnostic assessment. Triage is commonly used in healthcare for this reason [6]. See Table 5 for triage outcomes and actions. The triage team should include expert practitioners across the age range of different diagnoses and available local services (e.g., paediatrician, psychologist, OT). Frequency and length of meetings to process referrals depends on referral rate. To support the provision of high quality information prior to referral, a standardised information gathering approach is important, ensuring consistent understanding and documentation. It may be necessary for the team completing triage to complete more information gathering before to the first formal appointment (recommended tools are summarised in Table 6).

### 3.5. Which ‘Disorder Specific’ Assessment Tools Should Be Considered?

We identified that a neurodevelopmental assessment should include:

- medical and developmental history;
- presentation at current time-point;
- reports from the person/self-report/informant report;
- assessments from people in the environment of the person (e.g., home);
- clinical observation in different natural environments (e.g., home, school);
- assessment and skills;
- assessment of function and participation activities/interactions that are developmentally appropriate/relevant;
- the environment, including social and physical features around the child and family.

Several tools can be applied depending on presenting concerns (see Supplementary File S2 for a full list of tools, commonly used and recommended either in published clinical guidelines or by expert stakeholders). Clinical expertise and consultation across relevant teams can be used to identify the necessary assessments. The assessments can be completed together or separately. Assessments do not need to be completed in sequence, and may not be targeted to a specific presentation or diagnosis.

### 3.6. What Are the Key Considerations for Making a Diagnosis and Reporting This to Individuals and Their Families?

Information should be brought together and considered in a procedure termed ‘formulation’ [67]. This was a term familiar to some of our stakeholders, but not all. One benefit of a multi-disciplinary approach is the spread of shared language and practices. Formulation ideally takes account of ideas, thoughts and experiences of the individual and their family members in relation to diagnosis. Diagnoses are then made with regard to international criteria. The research team developed tables of evidence (see Supplementary File S3 for full tables of evidence for each diagnosis) to support clinicians in reviewing assessment evidence and reaching a diagnosis. The tables contain DSM-5 criteria for autism, ADHD, ID, DLD, DCD and FASD. Criteria for each are located in a matrix designed to allow clinicians to view and compare information gathered from different elements in one location. The tables were designed to facilitate a decision as to whether enough information was present to support making a diagnosis. If diagnosis is not possible, the tables may indicate aspects requiring further information/review.

Stakeholders identified that qualitative and experiential aspects of diagnostic processes were extremely important. People respond to diagnosis in a multitude of ways, and an approach that is personalised is recommended. Several opportunities for discussion should be provided in order that discussion can be completed adequately and clarifications can be made if required. However, people and their families maintain the option of both accepting and/or disclosing a diagnosis to others. Any issues, idiosyncrasies or behaviours might not necessarily be seen as troublesome to people and their families. There are also important factors related to time, and potential changes in people (and families) view of diagnosis over a lifetime. There are therefore instances where it is necessary to review a diagnosis. Relatedly, there is a need to have a process to seek out, access or improve new or existing supports across the lifespan, including anticipatory support in advance of major transitions.

See Table 7 for agreed standards for diagnosis and follow up.

### 3.7. What Time Standards Should Be Applied to a Neurodevelopmental Pathway?

We developed time standards as a benchmark to report against (summarised in Table 8). Waiting for diagnosis is commonly raised as a major concern for those seeking support. We identified few published or formal time standards for diagnostic assessment. Time standards were therefore developed through consideration of the similar standards for autism e.g., [38,41,68] and consensus views gained from stakeholders.

### 3.8. How Can We Involve Lived Experience in the Development of Pathways?

There is a need for the co-production and involvement of users of services, experts by experience or ‘patient-voices’ at all stages [9,69] based on the principle of ‘nothing about us without us’ [70]. When a diagnosis is made, a new, potentially supportive community of people with similar experiences is opened up. Diagnosis matters to people seeking and receiving assessment. However, the process is experienced differently, and sometimes it is positive, while at other times it may be challenging [14,71]. Based on our consultation with representatives of people with recent experience in diagnostic assessment, key points for reflection are summarised in Table 9 (and Supplementary File S4 for further detail).

## 4. Discussion

In this paper, we shared the process and outcomes in the development of a new pathway for neurodevelopmental assessment and diagnosis. Stakeholder engagement, along with review of current guidelines and assessment tools, allowed the development of recommendations that are feasible and evidence informed. In this article, we have outlined the reasons why a neurodevelopmental approach is desirable, together with the presentation of solutions and recommendations for practitioners and services undertaking this change.

The role of diagnosis may be controversial; however, a preference for timely diagnosis is well-supported in the literature [72,73,74]. We strongly advocate the perspective that people’s needs should be addressed irrespective of diagnosis or ‘label’. However, it is often the case that a diagnosis can help with accessing needed supports. In particular, when people are at key points in their lives, when they are accessing new environments, situations or changing circumstances, a diagnosis is helpful in clarifying how neurodevelopmental differences may be impacting an individual, and therefore, the interventions, supports or adaptations required. The need for evidence-informed intervention, particularly non-pharmacological interventions and environmental adaptations, could be made more explicit by a recognised diagnosis [3].

### 4.1. Implications for Practice

Key points identified from evidence and consensus are summarised below.

- Our research highlights the need for a neurodevelopmental understanding, rather than focusing on single conditions, in clinical assessment and diagnosis.
- Most individuals will have signs of more than one diagnosis; a co-occurring neurodevelopmental presentation is the norm.
- Diagnostic assessment has historically focused on individual presentations. In Scotland, many areas had a children’s autism or ADHD pathway/service. Based on these models, children and young people could wait on one list after another.
- Individual professionals may be highly knowledgeable or experienced in one ‘diagnosis’ but only have an ‘informed’ or ‘skilled’ level for others.
- Assessment and diagnosis services should be multi-disciplinary, people receiving help should be directed to the relevant professionals as required.
- As well as a diagnostic ‘label’, formulation should encompass the needs, wishes, strengths and goals of the individual, and focus on their day-to-day life and typical environments.
- Neurodevelopmental differences are lifelong, but need not be framed as ‘deficits’. Outcomes for individuals are strongly influenced by the environment, demands of activities, available resources and supports, and the individual’s own strengths and motivations (i.e., a social model of disability).
- Review and provision of pharmacological and non-pharmacological supports is required for interventions in relation to associated needs including sleep, anxiety, and mood.
- While accurate diagnosis can enhance intervention, assessments and planning, support should never be purely diagnosis-dependent.

### 4.2. Limitations

Our recommendations are based primarily on expert consensus, although evidence from guidelines has been utilised where possible, and a review of assessment tools undertaken. Recommendations are aimed at children and young people. Adults may share similar features, but their day to day contexts and roles are different. Although this study was completed in Scotland, the demographic and cultural similarities of Scotland to other countries means recommendations are likely to be transferable. There is an ongoing need for service configuration data, and an understanding of ratio and skill mix of staff recommended for a given population. Work is ongoing in Scotland via NAIT to develop this.

### 4.3. Conclusions

We have developed a practical resource for neurodevelopmental assessment and diagnosis. The recommendations reported here are supported by the Scottish Government. All materials are freely available and recommended for use by those with relevant skills and experience in undertaking neurodevelopmental assessment in children and young people aged 0–18 years.

## Figures and Tables

**Table 1:** Clinical guidelines used to develop the pathway.

	Scotland	International
Attention Deficit Hyperactivity Disorder	Guideline no longer applies and is superseded by NICE (UK) 2018 [39]	NICE (UK) (2018) [39]
Autism	SIGN (2016) [26]	New Zealand Ministries of Health and Education (2016) [40]
		NICE (UK) (2011) [12]
		Penner (2018) [41]
		Whitehouse (2018) [42]
Developmental Co-ordination Disorder	No Guideline	Blank (2019) [43]
Developmental Language Disorder	No Guideline	Bishop (2016) [44]
		Bishop (2017) [45]
		The Association for Child and Adolescent Mental Health (2021) [46]
Foetal Alcohol Spectrum Disorder	SIGN (2019) [47] SIGN (2019) [48]	
Intellectual Disability	No Guideline	British Psychological Society (2000) [49]
		British Psychological Society (2015) [50]
		MacKay (2009) [51]
		Tassé (2019) [52]
Neurodevelopental	No Guideline	Gillberg (2010) [13]
		Gillberg (2021) [3]

**Table 2:** Pre-Referral Tools Applicable to all Diagnoses. Adapted from published NAIT pathway [35].

Tool Summary and Reference	Respondent	Stage/Age
The ESSENCE-Q [53] is a one-page list of yes/no questions to identify areas of concern, with room for brief elaboration. A useful screening questionnaire to gather pre-referral information.	Professional Parent/Carer	Early years, Primary, and Secondary (0–18 years)
The Social Communication, Emotional Regulation Transactional Supports (SCERTS) Tools [54] offer a structured format, adapted to developmental stage (from non-verbal to conversational level) based on observation in naturally occurring environments. Particular focus on social communication, emotional regulation and transactional supports.	Professional with training	All ages and stages
Fife Neurodevelopmental Questionnaire [55] is a developmental history gathering form for use with parents, developed in Scotland, based on the ESSENCE-Q.	Professional	0–18 years
Strengths and Difficulties Questionnaire (SDQ) [56] is a brief emotional and behavioural questionnaire, completed by parents/professionals and older young people.	Professional Parent/Carer Young person (>11 years)	2–17 years
The Developmental and Well-Being Assessment (DAWBA) [57] comprises a set of interviews and questionnaires that can be done on a computer or face to face. Designed to gather information relevant to a range of DSM-5 diagnoses.	Professional Parent/Carer Young person (>11 years)	2–17 years
The Griffiths Assessment [58] is a standardised, observational, play-oriented measure for assessing the rate of development of neurodevelopmental skills.	Pediatrician Professional with training	0–6 years
The CIRCLE Early Years Stages Tool (0–5 years) [59] or the CIRCLE Participation Scale (5–18 years) [18] identify factors that support or interfere with participation in school life for children with additional support needs.	Education professional	Early years, Primary, and Secondary (0–18 years)
The School Participation Questionnaire (SPQ) [60] is a measure to support understanding of participation related factors, involvement and engagement of children with additional support needs in the school context.	Education professional	Primary (5–12 years)
The Child and Adolescent Intellectual Disability Screening Questionnaire (CAIDS-Q) [61] is a quick, easy and accurate way of identifying children/young people who are likely to have an intellectual disability.	Professional Parent/Carer	6–17 years
The Dimensions Tool [62] is an online tool providing personalised information to support a person’s well-being and mental health.	A parent/carer, an individual, GP or other professional can use the rating against dimensions related to health and wellbeing	>13 years
The Five to Fifteen Tool (FTF) The FTF 2–5 years and FTF 5–17 years [63] are standardized and validated questionnaires to gather clinical history relevant to the entire range of neurodevelopmental presentations and can be used aid in early detection, enabling further examination and intervention.	Parent/carer Education professional	2–17 years

**Table 3:** Standards for Neurodevelopmental Assessment. Adapted from the published NAIT pathway [35].

Neurodevelopmental assessment should (ideally) be conducted by an MDT and always by more than one person Assessment should follow a clinical guideline if available Those undertaking assessment should have the relevant level of training and experience Assessment should be identified as early as possible as ‘core’ or ‘complex’ ‘Core’–which can be completed by the team currently involved or other local team ‘Complex’–which should be completed by a team identified at triage, who have skills required and time allocated or training in additional standardised diagnostic tools and formulation across different diagnoses Core assessments should be completed in 1–2 appointments with as much information as possible gathered in advance of the 1st MDT appointment Complex assessments should be carried out by an MDT with expertise in the presenting of differential diagnoses or co-occurring presentations Local teams should consider sending relevant useful information to individuals and families prior to assessment, for example: ‘Information for you while waiting for your appointment’ ‘Neurodevelopmental information leaflets and website links’ ‘What to expect in an online or face to face appointment’ Reports should be written on the day of an appointment and shared as agreed within two weeks for: each assessment appointment * diagnostic outcome report Formulation and diagnosis should be made with explicit reference to diagnostic criteria (DSM5 or ICD-11) Time from acceptance of referral to first appointment should be no longer than 12 weeks Local areas should consider including time standards in local pathways

* It is noted that in some circumstances a single assessment may be carried out over more than one appointment.

**Table 4:** Complexity factors which may be used to identify ‘core’ and ‘complex’ cases. Adapted from published NAIT pathway [35].

Complexity Factors	Examples
Medical history	More than one co-occurring diagnosis
	Genetic or chromosomal abnormalities
	Other neurodevelopmental disorders
	Intellectual disability
	Attachment disorder
	Mental health condition
	Sensory impairment
	Poor regulation in one or more environments
	Adverse childhood experiences
Individuals who belong to groups where diagnosis is commonly delayed or overlooked	Females
	Individuals who are or have been looked after and accommodated
	Black and minority ethnic individuals
	Individuals who internalise, mask or camouflage or present with ‘mild’ symptoms
Family factors	Discrepant observations of needs across contexts (e.g., family and school)
	Different views within the family or between the individual, family members
	Neurodevelopmental disorders within the wider family Family literacy issues, communication or learning difficulties
	Lack of access to a person who can give a good quality history (e.g., where parent has a learning difficulty)
	Cultural differences
	Family languages other than English or other dominant local language
Clinical team	Lack of access to skills, experience and resources
	Recruitment and retention of necessary staff
	Rural and remote areas–the smaller team may be more prepared to meet most needs arising, but may be less able to respond to unusual situations
	Threshold for complexity will depend on experience of the team
Other environmental factors	Individuals who have needs well met by those around them may present with less obvious signs (this could add or reduce complexity)
	Difficulty in accessibility and travel to clinics
COVID-19	Potential to add significant complexity

The most ‘severe’ presentation is not the most complex and nor is the ‘milder’ presentation the most straightforward. The number of complexity factors may or may not add up to a complex case. In different contexts a particular presentation may or may not be complex depending on the clinical team.

**Table 5:** Triage outcomes and actions. Adapted from published NAIT pathway [35].

Triage Outcomes	
Outcome	Actions
Not accepted: adequate information shared and effective conversations with person raising concern	Continue to collaborate in the planning process for this individual as required (support according to needs identified) Communication with person making request explaining the decision Signposting and advice for presenting concerns
Not accepted: not enough information	Communication with person making request explaining the decision, requesting specific further information or assessment Signposting and advice for presenting concerns
Accepted: core pathway	Make initial formulation of possible differential diagnoses to trigger appropriate assessment and enlist the right professionals Request further assessments prior to next appointment Allocate appointments to complete assessment with professionals currently known to individual referred or local team, following receipt of recommended assessments Link family with relevant supports and provide information on processes Inform person making request of outcome
Accepted: complex pathway	Make initial formulation of possible differential diagnoses to trigger appropriate assessment. Enlist the right professional team Request any further assessments required prior to next appointment On receipt of requested further assessments, allocate appointments and identify relevant teams to take assessment forward with named clinician(s) identified. Link family with relevant supports and provide information on processes Inform person making request of outcome

Before triage, check that the following are available: (1) consent for referral, (2) referral form(s), (3) ESSENCE-Q or similar screener, (4) reports on: professional views, reasons for referral; early development and family history (including prenatal alcohol history); parent/carer views about strengths, concerns, expectations; young person’s views; Relevant reports/assessments (e.g., occupational therapy).

**Table 6:** Post-triage further information gathering tools, prior to the face-to-face appointment. Adapted from published NAIT pathway [35].

Details	Respondent	Age/Stage
A Neurodevelopmental-informed school or nursery observation can be used to gather information in a natural setting, especially when there have been discrepant contextual reports from home and school. Any age, when the observation can be made without the child/young person being aware or uncomfortable. May not be suitable for older children. Should be considered but is not essential.	A professional with enhanced or expert skills in diagnosis	All ages and stages
The Social Responsiveness Scale (SRS) [64] is standardised tool, and useful where social communication differences are highlighted, to provide contextual information and clarity over whether or not there are discrepancies between home and school.	Parent/carer Education professional Scored by health professional	Pre-school (0–5 years) School (5–18 years)
The Conners Scale [65] is a questionnaire that is used as a screening tool to understand whether the child or young person may benefit from further detailed ADHD assessment.	Parent/carer School Scored by health professional	6–18 years
Behavior Rating Inventory of Executive Function (BRIEF) [66] is a short questionnaire designed to assess executive function in different contexts. May be used with those with intellectual disabilities and attention disorders, traumatic brain injuries, neurodevelopmental, psychiatric, and medical conditions.	Parent/carer Education professional Scored by health professional	5–18 years

**Table 7:** Standards for diagnosis and follow up. Adapted from published NAIT pathway [35].

Write report on day diagnostic decision is made and share report with family promptly Follow local protocols for information sharing with the wider team Share a report with the wider team and family according to preferences and consent Provide training and support to all health professionals involved in the pathway in relation to ‘sharing difficult news’ Share locally agreed upon information on the day of diagnosis, relevant to the child and family attending Offer a ‘follow up’ appointment soon after the diagnosis to give the family an opportunity to ask further questions and hear more about local planning processes and sources of support [within eight weeks of diagnosis] Make training and information available for school staff and the wider team Staff should follow clear guidelines about recommended sources of national and local support and advocacy for children and young people with a range of needs Staff should follow clear local protocols about linking parents in with parent mediated interventions and information sessions Parent information sessions support and interventions should be offered before, during and after diagnosis. They should be adapted for: the age and stage of their child children with neurodevelopmental differences Families report they particularly value: Face to face meetings with health and education professionals with expertise in the ‘diagnosed’ presentations at this age and stage, and with professionals with up to date experience and understanding of the local planning process The opportunity to ask questions Information relevant to their family, including support to apply for benefits (where appropriate) Local support groups, parent information/education sessions and parent mediated interventions Access to recommended supports

**Table 8:** Time Standards. Adapted from published NAIT pathway [35].

Stage in Pathway	Time Standard
1. Pre-referral (initial information gathering) *	First appointment should be as soon as possible and no later than four weeks (establish consent to refer/ request assistance and consent to share information)
2. Request for neurodevelopmental assessment (time from request for neurodevelopmental assessment accepted to first appointment after triage)	No more than 12 weeks
3. Diagnostic assessment (first appointment to last appointment)	Up to six weeks (core cases) Up to 22 weeks (complex cases)
4. Diagnosis (last appointment to diagnosis made; may include consensus that no diagnosis is made or individual does not meet criteria)	Less than one week
5. Diagnostic outcome (decision made to diagnostic assessment outcome shared)	Less than one week
6. Total time (from request/referral accepted to diagnosis shared)	No more than 19 weeks (core cases) No more than 36 weeks (complex cases)
7. Follow up (meeting after diagnosis)	Within eight weeks of diagnosis shared

* Offer a first appointment to those who have been identified (e.g., by family doctor) as in need of a neurodevelopmental assessment. Each service or local area will have pre-existing standards for responding to initial or safeguarding concerns.

**Table 9:** Lived experience themes and reflections for professionals. Adapted from published NAIT pathway [35].

Theme	Reflections for Professionals
No supports should be diagnosis dependent	Does your service limit access to supports before diagnosis and if so how can this be addressed? Are supports offered before, during and after diagnosis developmentally relevant and individualised to meet particular needs? How well are health, education and other services working together? Are families potentially wasting time on attending programmes that are not right for them?
Quality and timing of information provided is more important than quantity	What information is shared? How is information shared with families? Is information accessible? Are there opportunities both on the day of diagnosis and after, for the individual and family to have conversations with professionals with suitable experience about the diagnosis and financial, health, educational or other supports?
Keep families and individuals informed at every stage	How clear and accessible is the information about diagnosis? How predictable is the assessment process? Do things happen at the time and in the way expected? Do different professionals give consistent information? How can the process be made more positive?
Make the request for assessment and support process clear, especially when the decision is made not to proceed	Are individuals/families clear about how to request assessment and are they supported to do so? Do they know why the assessment is proceeding or not? Have they been made aware that a further request can be made if circumstances change or they disagree with the decision? Is information provided and support in place to address concerns raised by individuals/families?
Parents don’t know what they don’t know	How can professionals support families to ask the right questions and focus on key information?
Help set expectations about waiting times and provide a point of contact	How well are shared expectations communicated with and understood by families? Do families feel informed? Are families signposted to relevant support and made aware of their rights? Do professionals have access to relevant, accurate information about waiting times and sources of support?
At assessment appointments	Reflect on feedback from those who attend appointments. Ask people about what worked well/could be better in advance of the appointment, at the appointment and after it. Ask about the quality of the environment in the ‘clinic’ setting. Ask about the quality of communication of professionals.

## Data Availability

Please contact the corresponding author for study data.

## Supplementary Materials

The following are available online at https://www.mdpi.com/article/10.3390/children8111033/s1, Supplementary File S1: Pathway; Supplementary File S2: Standardised Assessment Tools Recommended for Particular Presentations; Supplementary File S3: Summary Tables of Evidence for Each NDD Diagnosis; Supplementary File S4: Views from People with Lived Experience.
