# Supplementary material for: Development of a Pathway for Multidisciplinary Neurodevelopmental Assessment and Diagnosis in Children and Young People
Source: Children (Basel). 2021 Nov 11;8(11):1033. doi: 10.3390/children8111033 (PMC8617836; doi:10.3390/children8111033)
Supplement: Supplementary file 1 [file children-08-01033-s001.zip › children-1422056-supplementary.pdf]

Supplemental files

Contents

Supplementary File S1: Pathway 2
- Pathway overview 2
- Pathway diagram 3

Supplementary File S2: Standardised Assessment tools recommended for particular presentations 4
- Assessment tools: Autism Spectrum Disorder 4
- Assessment tools: ADHD 5
- Assessment tools: Global Developmental Delay and Intellectual Disability 6
- Assessment tools: Developmental Co-ordination Disorder 7
- Assessment tools: Developmental Language Disorder 8
- Assessment tools: Fetal Alcohol Spectrum Disorder 9

Supplementary File S3: summary Tables of Evidence for each NDD diagnosis 11
- Summary Table of Evidence: Autism Spectrum Disorder 12
- Summary Table of Evidence: Attention Deficit and Hyperactivity Disorder 14
- Summary Table of Evidence: Intellectual Disability 16
- Summary Table of Evidence: Developmental Language Disorder 18
- Summary Table of Evidence: Developmental Co-ordination Disorder 20
- Summary Table of Evidence: Fetal Alcohol Spectrum Disorder 22

Supplementary File S4: views from people with lived experience 24

Supplementary File S1: Pathway

Pathway overview

The Neurodevelopmental pathway outlined here, is based on the principles of proportionate support according to need and that no support should be diagnosis dependent.

At the same time, a good quality, accurate and timely diagnosis, which follows clinical guideline standards is currently reported to be supportive and desired by most individuals with neurodevelopmental disorders and their families.

Diagnostic assessment is desirable and beneficial for a range of reasons.

The pathway is designed to be multi-disciplinary, and implemented through a collaborative and integrated approach between Community Child Health, CAMHS, Allied Health Professionals and Education Professionals. In some areas other professional groups will be involved. The following pathway diagram represents a summary of the process, with guidance detailed more fully within the main paper

Following diagnosis – in an ideal scenario nothing much should change, as support should not depend on this, however, the new knowledge arising from the assessment process can lead to specific post diagnostic needs for information, signposting and support. Diagnosis being shared might indicate this is a good time to review support and needs. Support needs usually need to be revisited, as the manifestation of neurodevelopmental differences changes over time and through different transitions.

Local pathways, referencing this diagram, could include additional guidance and information about;

- Contact information
- Request for assistance processes
- Local planning processes
- Local sources of information and support provided at universal, targeted and specialist levels
- How information is shared with the people who need to know, including referrers, children and young people and their families seeking assistance, the multi-disciplinary team around the child or young person
- Example leaflets are provided on the NAIT website and these can be adapted for local use

Pathway diagram

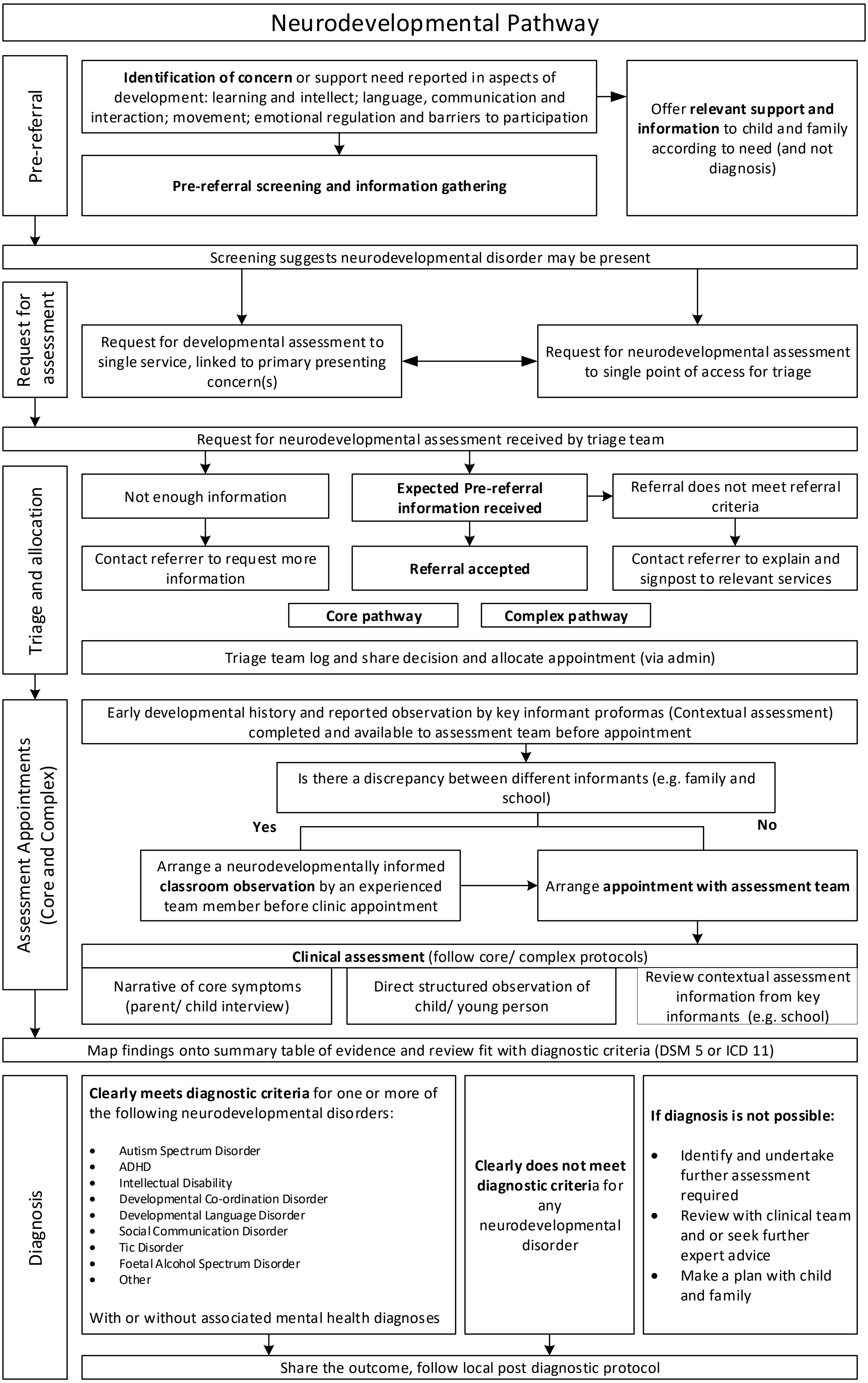

NAIT 2021, Children's Neurodevelopmental Pathway Available at: https://www.thirdspace.scot/nait/diagnosis-resources/

Supplementary File S2: Standardised Assessment tools recommended for particular presentations

Assessment tools: Autism Spectrum Disorder			
• It is not essential to use standardised autism tools in clinical diagnostic assessment of possible ASD<br>• Tools are used to standardise assessment of<br>• Reported history from individuals, parents or education staff<br>• Clinical observation<br>• Contextual assessment<br>• No diagnosis should be made based on one assessment tool outcome<br>• They are recommended in complex cases<br>• Training and experience in using these tools can improve clinical skills			
**Tool/ Assessment**	**Summary**	**Who administers/ scores the tool?**	**Age and stage**
**ADOS** (Autism Diagnostic Observation Schedule)	A standardised autism diagnostic observational assessment, with a diagnostic algorithm. The protocol consists of a series of structured and semi-structured tasks that involve social interaction between the examiner and the person under assessment, adapted to 5 different developmental levels.	ADOS trained practitioners	18 months to adulthood
**ADI-R** (Autism Diagnostic Interview-Revised)	A standardised, structured interview and response coding conducted with the parents of individuals who have been referred for the evaluation of possible autism or autism spectrum disorders.	ADI-R trained practitioners	Individuals at a developmental level of 2 years or above
**3DI** (Developmental, Dimensional and Diagnostic Interview)	A standardised, computer-based diagnostic interview for individuals with suspected ASD. It can be scored in terms of severity, frequency, and comorbidity.<br>Easy and practical in its use, requiring minimal training and automation of reporting.	3DI trained practitioners with access to computer with relevant software	• From age of 3 years<br>• From age 2 years with some modification<br>• For adolescents and adults with some modification of wording
**DISCO** (Diagnostic Interview for Social and Communication Disorders)	An interviewer-based schedule for use with parents and carers to obtain a developmental history and description of the child or adult concerned, where autism is being considered.	DISCO trained practitioners	• All ages and stages

Assessment tools: ADHD

- Standardised tools are commonly used but not essential in making ADHD diagnosis
- They are used as a structured way to collect information about concerns raised at home and school in relation to attention, activity and impulsivity
- These tools might be used at the pre-referral stage to support triage decisions

Tool/ Assessment	Summary	Who administers/ scores the tool?	Age and stage
**Conners 3 ADHD Rating Scale**	A short and long form scale to assess likelihood of ADHD. Can be used for screening or as a diagnostic tool.	a) Parent/carer<br>b) School<br>c) Self report by child<br>Scored by health professional	6-18 years
**SNAP IV**<br>Swanson, Nolan, and Pelham-IV Questionnaire	A screening tool for ADHD, which has high sensitivity but low specificity to clinical diagnosis	Teacher and Parent rating scale	6-18 years
**CADDRA**<br>The Canadian ADHD Resource Alliance	This organisation has a range of downloadable forms for adults with suspected ADHD to support assessment. These may also be relevant for adolescents (The WEISS; SNAP IV; the Adult ADHD Self Report Scale)		Adolescents and adults

Assessment tools: Global Developmental Delay and Intellectual Disability

- Tools are used to measure
  - Intellectual functioning
  - Adaptive function and impact on an individual's participation in everyday activities
- These can inform assessment where other neurodevelopmental diagnoses are being considered and where intellectual ability is important context in understanding the individuals needs and strengths
- They are not required for diagnosis of ID – (e.g. NHS Lothian have developed a pathway relevant across health and education)

Tool/ Assessment	Summary	Who administers/ scores the tool?	Age and stage
**Griffiths Assessment**	See main paper	Professionals trained in administration	Birth to 6 years
**WISC-V** (Wechsler Intelligence Scale for Children version 5)	A cognitive assessment/ intelligence test. It generates a Full Scale IQ score that represents a child's general intellectual ability. It also provides five primary index scores for different cognitive domains: Verbal Comprehension Index, Visual Spatial Index, Fluid Reasoning Index, Working Memory Index, and Processing Speed Index.	Psychologist	Age 6-16 years
**WPPSI-IV** (Wechsler Preshool and Primary Scale of Intelligence) – Fourth edition	A cognitive assessment / intelligence test for younger children.	Psychologist	2 years 6 months to 7 years, 7 months
**ABAS -3** (Adaptive Behavior Assessment System 3rd edition)	A rating scale useful for assessing skills of daily living. There is also an adult self-rating form.<br><br>The ABAS-3 covers three broad domains: conceptual, social, and practical, covering 11 skill areas. Tasks rated focus on everyday activities required to function, meet environmental demands, care for oneself, and interact with others effectively and independently.	Rating forms are filled out by the parent and a teacher<br><br>Scored by professionals	Birth to adulthood
**Vineland -3** (Vineland Adaptive Behavior Scales, Third Edition)	Semi-structured interview/ questionnaire to assess adaptive behaviour (Communication, Daily Living Skills, Socialisation, Motor Skills, Maladaptive behaviour).	Parent/ Carer<br>Teacher	Birth to adulthood

Assessment tools: Developmental Co-ordination Disorder

- A qualified Occupational Therapist (OT) can undertake assessment of development using a range of standardised and non-standardised tools and approaches to establish developmental level, understand the child/young person's profile and to inform planning and intervention for children and young people with suspected DCD
- Standardised tools to are commonly used assess
  - physical skills
  - and the functional impact of these
- In children with a learning/ intellectual disability, DCD should only be diagnosed if their physical co-ordination is significantly more impaired than their mental abilities
- Although DCD may be suspected in the pre-school years, it's not usually possible to make a definite diagnosis before a child is aged 4 or 5 years

Tool/ Assessment	Summary	Who administers/ scores the tool?	Age and stage
**Child Occupational Self-Assessment (COSA)**	A child directed assessment designed to capture children's perceptions regarding sense of competence and importance of participation in daily living activities.	Occupational Therapist trained in using the tool in collaboration with child	7-18 years
**Short Child Occupational Profile (SCOPE)**	An observational assessment that determines how child and environment factors facilitate or restrict participation in daily living activities.	Occupational Therapist trained in using the tool	Birth-21 years
**Movement ABC-2 (Movement Assessment Battery for Children)**	Used to identify a delay or impairment in motor development and whether there is a detrimental impact during activities at home and in school.	Usually an Occupational Therapist. May also be used by a Physiotherapist or Paediatrician	Full battery 3-16 years<br>Checklist 5-12 years
**Assessment of Motor and Process Skills (AMPS)**	An observational assessment used to assess performance quality in tasks related to daily living	Occupational Therapist trained in using the tool	3 years to adulthood

Assessment tools: Developmental Language Disorder

- An individual's language stage is important context for interpreting and selecting other relevant neurodevelopmental assessment tools or approaches
- Standardised tools are not essential for diagnosis of DLD but are usually used to support diagnosis
- A qualified Speech and Language Therapist (SLT) can undertake assessment of speech, and communication using a range of standardised and non-standardised tools and approaches to establish developmental level, understand the child/young person's language profile and to inform planning and intervention
- Assessment should consider receptive and expressive skills in: Phonology; Grammar (Syntax and Morphology); Verbal learning and memory; Word finding; Semantics (word meaning and vocabulary); Narrative and Pragmatics (Language Use)
- SLTs should follow departmental guidelines when carrying out assessment of the language and communication skills of bilingual learners. This is likely to involve informal and diagnostic assessment across languages, with support from Education services and/or an interpreter as appropriate
- Clinicians should consider the whole neurodevelopmental profile in interpreting assessments "These given difficulties are not a result of a sensory impairment, motor dysfunction or any other medical condition and cannot be attributed to intellectual disability or global developmental delay" (DSM 5)

Tool/ Assessment	Summary	Who administers/ scores the tool?	Age and stage
**CELF-5 UK** (Clinical Evaluation of Language Fundamentals - Fifth Edition)	Battery of 14 subtests giving standardised scores<br>• Index language scores for: core, receptive, expressive, content, structure and memory<br>• Observation Rating Scale for evaluation of language in context<br>• Pragmatics Profile and Pragmatic Activities Checklist to assess social communication skills	Speech and Language Therapist	5 years to 21 years 11 months
**CELF-Preschool 2 UK**	A standardised battery of subtests to measure a broad range of expressive and receptive language skills in young children.	Speech and Language Therapist	3-6 years
**The Pragmatics profile of everyday communication skills in children**	Non standardised questionnaire linked to developmental expectations of social pragmatic development. http://complexneeds.org.uk/modules/Module-2.4-Assessment-monitoring-and-evaluation/All/downloads/m08p080c/the_pragmatics_profile.pdf	Speech and Language Therapist	6 months and primary school age

Assessment tools: Fetal Alcohol Spectrum Disorder

Standardised Tools are recommended where possible to elicit significant difficulties in multiple brain areas. The following list is not exhaustive, and aims to give suggestions of tests which could be utilized as per SIGN guideline (156) spanning cognition, adaptive function, academic function, sensory / motor, language, memory and executive function. Measures of affect regulation should also indicate significant clinical impairment.

There may be circumstances where use of indirect assessment methods are more appropriate. In this case, clinicians should seek information from multiple sources e.g. use of screening instruments, direct observations and clinical interview. Judgement as to whether a domain should be at a severity level equating to <2 Standard Deviations should ideally take place within a multidisciplinary team discussion.

Tool/ Assessment	Summary	Who administers/ scores the tool?	Age and stage
**Griffiths**	See main paper		Birth to 6 years
**WPPSI-IV** (Wechsler Preshool and Primary Scale of Intelligence) – Fourth edition	See above in section on Global Developmental Delay and Intellectual Disability	Psychologist	2 years 6 months to 7 years, 7 months
**WISC-V** (Wechsler Intelligence Scale for Children version 5)	See above in section on Global Developmental Delay and Intellectual Disability	Psychologist	Age 6-16 years
**ABAS -3** (Adaptive Behavior Assessment System 3rd edition)	See above in section on Global Developmental Delay and Intellectual Disability	Parent/ Carer<br>Scored by Psychologist	Birth to adulthood
**Vineland -3** (Vineland Adaptive Behavior Scales, Third Edition)	See above in section on Global Developmental Delay and Intellectual Disability	Parent/ Carer version<br>Teacher version<br>Scored by trained professionals	Birth to adulthood
**CELF-5 UK** (Clinical Evaluation of Language Fundamentals - Fifth Edition)	See above section on Developmental Language Disorder	Speech and Language Therapist	5 years to 21 years 11 months
**Movement ABC-2** (Movement Assessment Battery for Children)	See above section on Developmental Co-ordination Disorder	Usually an Occupational Therapist. May also be used by a Physiotherapist or Paediatrician	Full battery 3-16 years<br>Checklist 5-12 years
**BEERY VMI** (Beery-Buktenica Developmental Test of Visual – Motor Integration)	Assesses visual-motor skills in children and adults.	Occupational Therapist	2-100 years
**Sensory Processing Measure**	Obtains a picture of sensory functioning at home, school and in the community	Occupational Therapist	
**WIAT-III –** (Wechsler Individual	Assesses reading, language and numerical attainment.	Psychologist	4 – 25 years 11 months

Achievement Test – Third Edition)			
**BRIEF** (Brief Rating Inventory of Executive Function)	Assesses executive function in children and adolescents	Usually a Psychologist	5 – 18 years
**BADS** (Behavioural Assessment of Dysexecutive Syndrome in Children)	Assessment of a number of aspects of executive function such as inflexibility and perseveration, impulsivity, planning and novel problem solving.	Psychologist	7-16 years
**CMS** Children's Memory Scale	Assessment of various memory functions	Psychologist	5 – 16 years
**Conners 3 ADHD Rating Scale**	See above section on ADHD	Parent/carer<br>School<br>Self report<br>Scored by health professional	6-18 years
**Test of Everyday Attention in Children – 2nd edition**	Standardised assessment of separate aspects of attention in children	Psychologist	5 – 15 years

Supplementary File S3: summary Tables of Evidence for each NDD diagnosis

- Autism Spectrum Disorder
- Attention Deficit and Hyperactivity Disorder
- Intellectual Disability
- Developmental Language Disorder
- Developmental Co-ordination Disorder
- Fetal Alcohol Spectrum Disorder

This document is designed for clinical purposes and use by experienced diagnostic teams

A note on how to use the tables of evidence:
Diagnosis involves taking a complex set of observations and information from a range of sources over time and considering this in relation to the agreed diagnostic criteria. Although algorithms and consistent descriptors are intended to make the process more reliable and robust, diagnosis can be as much of an art as a science and high levels of clinician expertise increases reliability.

How can they be used?

- These tables of evidence are intended as a summary tool for the clinical team, to support new and experienced practitioners to become familiar with diagnostic criteria and to allow discussion between team members about evidence gathered and what it means
- They can be used to confirm whether there are discrepancies or agreements between different sources of information
- They can help identify gaps in information required
- Within a neurodevelopmental pathway, they may highlight the need to involve another professional in the team around the child
- A tick in a box should indicate that due consideration has been given to developmental and contextual relevance of examples of observed signs and symptoms (e.g. quality, frequency, recency)

What limitations are there?

- They should not replace the complex and nuanced clinical reasoning of expert practitioners in the multi-disciplinary team
- They should not be used as a list to tick every time you see one of these signs until there is enough for a diagnosis

Summary Table of Evidence: Autism Spectrum Disorder

Summary Table of Evidence: Autism Spectrum Disorder

Guidance Notes

- The *Summary Table of Evidence* provides an efficient way of recording and presenting information obtained through assessment
- It has been designed to allow clinicians to view and compare information gathered from different elements of assessment, on one page, in order to identify 'at a glance' whether there is enough evidence to support a particular diagnosis, combination of diagnoses or not
- In addition, if diagnosis is not possible, the *Summary Table of Evidence*, may indicate which aspect of the 4 domains is lacking information. See pathway for assessment options
- The table can be completed by a diagnostician individually, although preferably it would be considered by more than one person
- To complete the form, use completed assessment tools, reports and proformas. Transfer the ratings given onto the *Summary Table of Evidence* using the key overleaf
- Once completed, this form should provide a summary of evidence relating to each of the 4 diagnostic domains of DSM 5* diagnostic criteria

*American Psychiatric Association. (2013). *Diagnostic and statistical manual of mental disorders (DSM-5®)*. American Psychiatric Pub.

DSM 5 requires the following for diagnosis of Autism Spectrum Disorder

Must meet criteria A,B,C,D

A. Persistent deficits in social communication and social interaction across multiple contexts, as manifested by the following, currently or by history (examples are illustrative, not exhaustive, see text):

1. Deficits in social-emotional reciprocity, ranging, for example, from abnormal social approach and failure of normal back-and-forth conversation; to reduced sharing of interests, emotions, or affect; to failure to initiate or respond to social interactions.
2. Deficits in nonverbal communicative behaviours used for social interaction, ranging, for example, from poorly integrated verbal and nonverbal communication; to abnormalities in eye contact and body language or deficits in understanding and use of gestures; to a total lack of facial expressions and nonverbal communication.
3. Deficits in developing, maintaining, and understanding relationships, ranging, for example, from difficulties adjusting behaviour to suit various social contexts; to difficulties in sharing imaginative play or in making friends; to absence of interest in peers.

Specify current severity: Severity is based on social communication impairments and restricted repetitive patterns of behaviour. (See table below.)

B. Restricted, repetitive patterns of behaviour, interests, or activities, as manifested by at least two of the following, currently or by history (examples are illustrative, not exhaustive; see text):

1. Stereotyped or repetitive motor movements, use of objects, or speech (e.g., simple motor stereotypies, lining up toys or flipping objects, echolalia, idiosyncratic phrases).
2. Insistence on sameness, inflexible adherence to routines, or ritualized patterns or verbal nonverbal behaviour (e.g., extreme distress at small changes, difficulties with transitions, rigid thinking patterns, greeting rituals, need to take same route or eat food every day).
3. Highly restricted, fixated interests that are abnormal in intensity or focus (e.g., strong attachment to or preoccupation with unusual objects, excessively circumscribed or perseverative interest).
4. Hyper- or hyporeactivity to sensory input or unusual interests in sensory aspects of the environment (e.g., apparent indifference to pain/temperature, adverse response to specific sounds or textures, excessive smelling or touching of objects, visual fascination with lights or movement).

Specify current severity: Severity is based on social communication impairments and restricted, repetitive patterns of behaviour. (See table below.)

C. Symptoms must be present in the early developmental period (but may not become fully manifest until social demands exceed limited capacities or may be masked by learned strategies in later life).

D. Symptoms cause clinically significant impairment in social, occupational, or other important areas of current functioning.

E. These disturbances are not better explained by intellectual disability (intellectual developmental disorder) or global developmental delay. Intellectual disability and autism spectrum disorder frequently co-occur; to make comorbid diagnoses of autism spectrum disorder and intellectual disability, social communication should be below that expected for general developmental level.

Note: Individuals with a well-established DSM-IV diagnosis of autistic disorder, Asperger's disorder, or pervasive developmental disorder not otherwise specified should be given the diagnosis of autism spectrum disorder. Individuals who have marked deficits in social communication, but whose symptoms do not otherwise meet criteria for autism spectrum disorder, should be evaluated for social (pragmatic) communication disorder.

Specify if:

- With or without accompanying intellectual impairment
- With or without accompanying language impairment

Autism Spectrum Disorder

Child's name:
Date of birth:
Contact details/ CHI:
Date completed:
Completed by:

Key: Is this an area of challenge?
4 *None of the time*
3 *Some of the time*
2 *Most of the time*
1 *All of the time*

			PRIOR TO FIRST APPOINTMENT										FIRST APPOINTMENT				
**DSM Criteria**	**Diagnostic Areas**	**Diagnostic Items**	Early Developmental History		Reported observation by key informant (1)				Reported observation by key informant (2)				Narrative of Core Symptoms		Direct Observation by practitioner		MEETS DIAGNOSTIC CRITERIA
A*	Interaction	a. social approach	Y	N	4	3	2	1	4	3	2	1	Y	N	Y	N	
		b. two way interaction	Y	N	4	3	2	1	4	3	2	1	Y	N	Y	N	
		c. interest in others	Y	N	4	3	2	1	4	3	2	1	Y	N	Y	N	
	Nonverbal	a. verbal/ nonverbal integration	Y	N	4	3	2	1	4	3	2	1	Y	N	Y	N	
		b. using nonverbal communication (NVC)	Y	N	4	3	2	1	4	3	2	1	Y	N	Y	N	
		c. understanding others NVC	Y	N	4	3	2	1	4	3	2	1	Y	N	Y	N	
	Relationships	a. adjusting behaviour	Y	N	4	3	2	1	4	3	2	1	Y	N	Y	N	
		b. imaginative play/ activities	Y	N	4	3	2	1	4	3	2	1	Y	N	Y	N	
		c. quality of friendships	Y	N	4	3	2	1	4	3	2	1	Y	N	Y	N	
B*	Stereotyped or repetitive behaviour	a. motor stereotypies	Y	N	4	3	2	1	4	3	2	1	Y	N	Y	N	
		b. uses objects repetitively	Y	N	4	3	2	1	4	3	2	1	Y	N	Y	N	
		c. repetitive use of language	Y	N	4	3	2	1	4	3	2	1	Y	N	Y	N	
	Adherence to routines	a. motor rituals	Y	N	4	3	2	1	4	3	2	1	Y	N	Y	N	
		b. preference for sameness	Y	N	4	3	2	1	4	3	2	1	Y	N	Y	N	
		c. reactions to change	Y	N	4	3	2	1	4	3	2	1	Y	N	Y	N	
	Restricted interests	a. fixations	Y	N	4	3	2	1	4	3	2	1	Y	N	Y	N	
		b. attachment/ pre-occupation	Y	N	4	3	2	1	4	3	2	1	Y	N	Y	N	
		c. circumscribed/ pervasive	Y	N	4	3	2	1	4	3	2	1	Y	N	Y	N	
	Hyper or Hypo reactivity to sensory input	a. indifference to pain/cold/heat	Y	N	4	3	2	1	4	3	2	1	Y	N	Y	N	
		b. response to sounds/ textures	Y	N	4	3	2	1	4	3	2	1	Y	N	Y	N	
		c. fascination with sensory aspects of materials (e.g. spinning/ touching)	Y	N	4	3	2	1	4	3	2	1	Y	N	Y	N	
C*	Early Indicators	a. differences/ difficulties in childhood	Y	N													
		b. family history of ASD	Y	N													
		c. risk factors for ASD present	Y	N													
D**	Social, occupational, or other areas of current functioning	a. self-care (e.g. eating, drinking, sleep, hygiene)	Y	N	4	3	2	1	4	3	2	1	Y	N			
		b. productivity (household routines, school, work, community, engaging in family activities)	Y	N	4	3	2	1	4	3	2	1	Y	N			
		c. Community life (scheduled or unscheduled community activities, recreation/ leisure)	Y	N	4	3	2	1	4	3	2	1	Y	N			
E*	Intellectual disability	a. evidence of intellectual disability	Y	N	4	3	2	1	4	3	2	1	Y	N	Y	N	
		b. evidence of language impairment	Y	N	4	3	2	1	4	3	2	1	Y	N	Y	N	

*American Psychiatric Association. (2013). *Diagnostic and statistical manual of mental disorders (DSM-5®)*. American Psychiatric Pub.
**Headings to address Criterion D, taken from World Health Organisation (2013) International Classification of Functioning. Available at https://www.who.int/classifications/drafticfpracticalmanual.pdf accessed 21.02.2020

Outcome

◯ **Meets diagnostic criteria** ◯ **Does not meet diagnostic criteria** ◯ **Diagnosis unclear;** undertake further assessment or review in ___ months

Summary Table of Evidence: Attention Deficit and Hyperactivity Disorder

Summary Table of Evidence: Attention Deficit and Hyperactivity Disorder

Guidance Notes

- The *Summary Table of Evidence* provides an efficient way of recording and presenting information obtained through assessment
- It has been designed to allow clinicians to view and compare information gathered from different elements of assessment, on one page, in order to identify 'at a glance' whether there is enough evidence to support a particular diagnosis, combination of diagnoses or not
- In addition, if diagnosis is not possible, the Summary Table of Evidence, may indicate which aspect of the assessment domains is lacking information. See pathway for assessment options
- The table can be completed by a diagnostician individually, although preferably it would be considered by more than one person
- To complete the form, use completed assessment tools, reports and proformas. Transfer the ratings given onto the Summary Table of Evidence using the key overleaf
- Once completed, this form should provide a summary of evidence relating to each of the diagnostic domains of DSM 5* diagnostic criteria

*American Psychiatric Association. (2013). *Diagnostic and statistical manual of mental disorders (DSM-5®)*. American Psychiatric Pub.

DSM 5 requires the following for diagnosis of Attention Deficit and Hyperactivity Disorder

A. **A persistent pattern of inattention and/or hyperactivity–impulsivity that interferes with functioning or development as characterised by (1) and (2) below:**

(1) Inattention: Six or more symptoms of inattention for children up to age 16 years, or five or more for adolescents age 17 years and older and adults; symptoms of inattention have been present for at least 6 months, and they are inappropriate for developmental level:

a. Often fails to give close attention to details or makes careless mistakes in schoolwork, at work, or with other activities.
b. Often has trouble holding attention on tasks or play activities.
c. Often does not seem to listen when spoken to directly.
d. Often does not follow through on instructions and fails to finish schoolwork, chores, or duties in the workplace (e.g., loses focus, side-tracked).
e. Often has trouble organizing tasks and activities.
f. Often avoids, dislikes, or is reluctant to do tasks that require mental effort over a long period of time (such as schoolwork or homework).
g. Often loses things necessary for tasks and activities (e.g. school materials, pencils, books, tools, wallets, keys, paperwork, eyeglasses, mobile telephones).
h. Is often easily distracted
i. Is often forgetful in daily activities.

(2) Hyperactivity and Impulsivity: Six or more symptoms of hyperactivity-impulsivity for children up to age 16 years, or five or more for adolescents age 17 years and older and adults; symptoms of hyperactivity-impulsivity have been present for at least 6 months to an extent that is disruptive and inappropriate for the person's developmental level:

a. Often fidgets with or taps hands or feet, or squirms in seat.
b. Often leaves seat in situations when remaining seated is expected.
c. Often runs about or climbs in situations where it is not appropriate (adolescents or adults may be limited to feeling restless).
d. Often unable to play or take part in leisure activities quietly.
e. Is often "on the go" acting as if "driven by a motor".
f. Often talks excessively.
g. Often blurts out an answer before a question has been completed.
h. Often has trouble waiting their turn.
i. Often interrupts or intrudes on others (e.g., butts into conversations or games)

In addition, the following conditions must be met:

B. Several inattentive or hyperactive-impulsive symptoms were present before age 12 years.
C. Several symptoms are present in two or more settings, (such as at home, school or work; with friends or relatives; in other activities).
D. There is clear evidence that the symptoms interfere with, or reduce the quality of, social, school, or work functioning.
E. The symptoms are not better explained by another mental disorder (such as a mood disorder, anxiety disorder, dissociative disorder, or a personality disorder). The symptoms do not happen only during the course of schizophrenia or another psychotic disorder.

Based on the types of symptoms, three kinds (presentations) of ADHD can be specified:

- *Combined Presentation*: if enough symptoms of both criteria inattention and hyperactivity-impulsivity were present for the past 6 months
- *Predominantly Inattentive Presentation*: if enough symptoms of inattention, but not hyperactivity-impulsivity, were present for the past six months
- *Predominantly Hyperactive-Impulsive Presentation*: if enough symptoms of hyperactivity-impulsivity, but not inattention, were present for the past six months.

Because symptoms can change over time, the presentation may also change over time

ADHD

Child's name:
Date of birth:
Contact details/ CHI:
Date completed:
Completed by:

Key: Is this an area of challenge?
***4** None of the time*
***3** Some of the time*
***2** Most of the time*
***1** All of the time*

*American Psychiatric Association. (2013). *Diagnostic and statistical manual of mental disorders (DSM-5®)*. American Psychiatric Pub.

		PRIOR TO FIRST APPOINTMENT										FIRST APPOINTMENT				MEETS DIAGNOSTIC CRITERIA
**DSM Criteria**	**Diagnostic Items**	Early Developmental History		Reported observation by key informant and /or i ndividual				Reported observation by key informant from education setting				Narrative of Core Symptoms		Direct Observation by practitioner		
**A. Inattention**: Six or more symptoms of inattention for children up to age 16 years, or five or more for adolescents age 17 years and older and adults; symptoms of inattention have been present for at least 6 months, and they are inappropriate for developmental level:		Y	N	4	3	2	1	4	3	2	1	Y	N	Y	N	
(1)	1. Often fails to give close attention to details or makes careless mistakes in schoolwork, at work, or with other activities.	Y	N	4	3	2	1	4	3	2	1	Y	N	Y	N	
	2. Often has trouble holding attention on tasks or in play.	Y	N	4	3	2	1	4	3	2	1	Y	N	Y	N	
	3. Often does not seem to listen when spoken to directly.	Y	N	4	3	2	1	4	3	2	1	Y	N	Y	N	
	4. Often does not follow through on instructions and fails to finish schoolwork, chores, or duties in the workplace (e.g., loses focus, side-tracked).	Y	N	4	3	2	1	4	3	2	1	Y	N	Y	N	
	5. Often has trouble organizing tasks and activities.	Y	N	4	3	2	1	4	3	2	1	Y	N	Y	N	
	6. Often avoids, dislikes, or is reluctant to do tasks that require mental effort over a long period of time (such as schoolwork, homework).	Y	N	4	3	2	1	4	3	2	1	Y	N	Y	N	
	7. Often loses things necessary for tasks and activities (e.g. school materials, pencils, books, tools, wallets, keys, paperwork, eyeglasses, mobile telephones).	Y	N	4	3	2	1	4	3	2	1	Y	N	Y	N	
	8. Is often easily distracted	Y	N	4	3	2	1	4	3	2	1	Y	N	Y	N	
	9. Is often forgetful in daily activities.	Y	N	4	3	2	1	4	3	2	1	Y	N	Y	N	
(2)	a. Often fidgets with or taps hands or feet, or squirms in seat.	Y	N	4	3	2	1	4	3	2	1	Y	N	Y	N	
	b. Often leaves seat in situations when remaining seated is expected.	Y	N	4	3	2	1	4	3	2	1	Y	N	Y	N	
	c. Often runs about or climbs in situations where it is not appropriate (adolescents or adults may be limited to feeling restless).	Y	N	4	3	2	1	4	3	2	1	Y	N	Y	N	
	d. Often unable to play or take part in leisure activities quietly.	Y	N	4	3	2	1	4	3	2	1	Y	N	Y	N	
	e. Is often "on the go" acting as if "driven by a motor"	Y	N	4	3	2	1	4	3	2	1	Y	N	Y	N	
	f. Often talks excessively.	Y	N	4	3	2	1	4	3	2	1	Y	N	Y	N	
	g. Often blurts out an answer before a question is completed.	Y	N	4	3	2	1	4	3	2	1	Y	N	Y	N	
	h. Often has trouble waiting their turn.	Y	N	4	3	2	1	4	3	2	1	Y	N	Y	N	
	i. Often interrupts or intrudes on others (e.g., butts into conversations or games)	Y	N	4	3	2	1	4	3	2	1	Y	N	Y	N	
**B.** Several inattentive or hyperactive-impulsive symptoms were present before age 12 years.		Y	N	4	3	2	1	4	3	2	1	Y	N	Y	N	
**C.** Several symptoms are present in two or more settings, (such as at home, school or work; with friends or relatives; in other activities).		Y	N	4	3	2	1	4	3	2	1	Y	N	Y	N	
**D.** There is clear evidence that the symptoms interfere with, or reduce the quality of, social, school, or work functioning.		Y	N	4	3	2	1	4	3	2	1	Y	N	Y	N	
**E.** The symptoms are not better explained by another mental disorder (e.g.mood disorder, anxiety disorder, dissociative disorder, or personality disorder). Symptoms do not happen only during course of schizophrenia or another psychotic disorder.		Y	N	4	3	2	1	4	3	2	1	Y	N	Y	N	

Outcome

◯ **Meets diagnostic criteria** ◯ **Does not meet diagnostic criteria** ◯ **Diagnosis unclear;** undertake further assessment or review in ___ months

Presentation is: ◯ **Combined** ◯ **Predominantly inattentive** ◯**Predominantly hyperactive-impulsive**

Summary Table of Evidence: Intellectual Disability

Summary Table of Evidence: Intellectual Disability
**Guidance Notes**

- The *Summary Table of Evidence* provides an efficient way of recording and presenting information obtained through assessment
- It has been designed to allow clinicians to view and compare information gathered from different elements of assessment, on one page, in order to identify 'at a glance' whether there is enough evidence to support a particular diagnosis, combination of diagnoses or not
- In addition, if diagnosis is not possible, the Summary Table of Evidence, may indicate which aspect of the assessment domains is lacking information. See pathway for assessment options
- The table can be completed by a diagnostician individually, although preferably it would be considered by more than one person
- To complete the form, use completed assessment tools, reports and proformas. Transfer the ratings given onto the Summary Table of Evidence using the key overleaf
- Once completed, this form should provide a summary of evidence relating to each of the diagnostic domains of DSM 5* diagnostic criteria

*American Psychiatric Association. (2013). *Diagnostic and statistical manual of mental disorders (DSM-5®)*. American Psychiatric Pub.

DSM 5 requires the following for diagnosis of Intellectual Disability

Intellectual Disability (Intellectual Developmental Disorder) is a disorder with onset during the developmental period that includes intellectual and adaptive functioning deficits in conceptual, social and practical domains. The following three criteria must be met:

A. Deficits in intellectual functioning, such as reasoning, problem solving, planning, abstract thinking, judgment, academic learning, and learning from experience confirmed by clinical evaluation and individualized standard intelligence testing

B. Deficits in adaptive functioning that result in failure to meet developmental and sociocultural standards for personal independence and social responsibility. Without ongoing support, the adaptive deficits limit functioning in one or more activities of daily life, such as communication, social participation and independent living across multiple environment as such as home, school, work and community

C. Onset of these development and adaptive deficits during the developmental period

DSM 5 guidance on Global Developmental Delay

This diagnosis is reserved for individuals under the age of 5 years when the clinical severity level cannot be reliably assessed during early childhood. This category is diagnosed when an individual fails to meet expected developmental milestones in several areas of intellectual functioning, and applies to individuals who are unable to undergo systemic assessments of intellectual functioning including children who are too young to participate in standardised testing. This category requires re-assessment after a period of time.

Intellectual Disability

Child's name:
Date of birth:
Contact details/ CHI:
Date completed:
Completed by:

Key: Is this an area of challenge?
***4** None of the time*
***3** Some of the time*
***2** Most of the time*
***1** All of the time*

*American Psychiatric Association. (2013). *Diagnostic and statistical manual of mental disorders (DSM-5®)*. American Psychiatric Pub.

		PRIOR TO FIRST APPOINTMENT										FIRST APPOINTMENT				
**DSM Criteria**	**Diagnostic Items**	Early Developmental History		Assessment tools focussed on individual cognitive profiles				Reported observation by key informant from education setting				Narrative of Core Symptoms		Direct Observation by practitioner		MEETS DIAGNOSTIC CRITERIA
Intellectual Disability (Intellectual Developmental Disorder) is a disorder with onset during the developmental period that includes intellectual and adaptive functioning deficits in conceptual, social and practical domains. The following three criteria must be met:		Y	N	4	3	2	1	4	3	2	1	Y	N	Y	N	
A	Deficits in intellectual functioning, such as reasoning, problem solving, planning, abstract thinking, judgment, academic learning, and learning from experience confirmed by clinical evaluation and individualized standard intelligence testing	Y	N	4	3	2	1	4	3	2	1	Y	N	Y	N	
**B**	Deficits in adaptive functioning that result in failure to meet developmental and sociocultural standards for personal independence and social responsibility. Without ongoing support, the adaptive deficits limit functioning in one or more activities of daily life, such as communication, social participation and independent living across multiple environment as such as home, school, work and community	Y	N	4	3	2	1	4	3	2	1	Y	N	Y	N	
C	Onset of these development and adaptive deficits during the developmental period	Y	N	4	3	2	1	4	3	2	1	Y	N	Y	N	

Outcome

◯ **Meets diagnostic criteria** ◯ **Does not meet diagnostic criteria** ◯ **Diagnosis unclear;** undertake further assessment or review in ___ months

◯ **Meets criteria for Global Developmental Delay**

Summary Table of Evidence: Developmental Language Disorder

Summary Table of Evidence: Developmental Language Disorder
**Guidance Notes**
• The *Summary Table of Evidence* provides an efficient way of recording and presenting information obtained through assessment<br>• It has been designed to allow clinicians to view and compare information gathered from different elements of assessment, on one page, in order to identify 'at a glance' whether there is enough evidence to support a particular diagnosis, combination of diagnoses or not<br>• In addition, if diagnosis is not possible, the Summary Table of Evidence, may indicate which aspect of the assessment domains is lacking information. See pathway for assessment options<br>• The table can be completed by a diagnostician individually, although preferably it would be considered by more than one person<br>• To complete the form, use completed assessment tools, reports and proformas. Transfer the ratings given onto the Summary Table of Evidence using the key overleaf<br>• Once completed, this form should provide a summary of evidence relating to each of the diagnostic domains of DSM 5* diagnostic criteria<br><br>*American Psychiatric Association. (2013). *Diagnostic and statistical manual of mental disorders (DSM-5®)*. American Psychiatric Pub.
**DSM 5 requires the following for diagnosis of Developmental Language Disorder**
The following criteria should be present:<br><br>A. Persistent difficulties in the acquisition and use of language across modalities (i.e. spoken, written, sign language or other) due to deficits in comprehension or production that include the following:<br><br>1. Reduced vocabulary (word knowledge and use).<br>2. Limited sentence structure (ability to put words and word endings together to form sentences based on the rules of grammar and morphology).<br>3. Impairments in discourse (ability to use vocabulary and connect sentences to explain or describe a topic or series of events or have a conversation).<br><br>B. Language abilities are substantially and quantifiably below those expected for age, resulting in functional limitations in effective communication, social participation, academic achievement, or occupational performance individually or in any combination<br><br>C. Onset of symptoms is in the early developmental period<br><br>D. The difficulties are not attributable to hearing or other sensory impairment, motor dysfunction or another medical or neurological condition and are not better explained by intellectual disability, intellectual developmental disorder or global developmental delay

Developmental Language Disorder

Child's name:
Date of birth:
Contact details/ CHI:
Date completed:
Completed by:

Key: Is this an area of challenge?
***4** None of the time*
***3** Some of the time*
***2** Most of the time*
***1** All of the time*

*American Psychiatric Association. (2013). *Diagnostic and statistical manual of mental disorders (DSM-5®)*. American Psychiatric Pub.

DSM Criteria	Diagnostic Items	PRIOR TO FIRST APPOINTMENT: Early Developmental History		PRIOR TO FIRST APPOINTMENT: Speech, Language and communication assessment				PRIOR TO FIRST APPOINTMENT: Reported observation by key informant from education setting				FIRST APPOINTMENT: Narrative of Core Symptoms		FIRST APPOINTMENT: Direct Observation by practitioner		MEETS DIAGNOSTIC CRITERIA
A.	Persistent difficulties in the acquisition and use of language across modalities (i.e. spoken, written, sign language or other) due to deficits in comprehension or production that include the following:	Y	N	4	3	2	1	4	3	2	1	Y	N	Y	N	
	1. Reduced vocabulary (word knowledge and use).	Y	N	4	3	2	1	4	3	2	1	Y	N	Y	N	
	2. Limited sentence structure (ability to put words and word endings together to form sentences based on the rules of grammar and morphology).	Y	N	4	3	2	1	4	3	2	1	Y	N	Y	N	
	3. Impairments in discourse (ability to use vocabulary and connect sentences to explain or describe a topic or series of events or have a conversation).	Y	N	4	3	2	1	4	3	2	1	Y	N	Y	N	
B.	Language abilities are substantially and quantifiably below those expected for age, resulting in functional limitations in effective communication, social participation, academic achievement, or occupational performance individually or in any combination	Y	N	4	3	2	1	4	3	2	1	Y	N	Y	N	
C.	Onset of symptoms is in the early developmental period	Y	N	4	3	2	1	4	3	2	1	Y	N	Y	N	
D.	The difficulties are not attributable to hearing or other sensory impairment, motor dysfunction or another medical or neurological condition and are not better explained by intellectual disability, intellectual developmental disorder or global developmental delay	Y	N	4	3	2	1	4	3	2	1	Y	N	Y	N	

Outcome

◯ **Meets diagnostic criteria** ◯ **Does not meet diagnostic criteria** ◯ **Diagnosis unclear;** undertake further assessment or review in ___ months

Summary Table of Evidence: Developmental Co-ordination Disorder

Summary Table of Evidence: Developmental Co-ordination Disorder
**Guidance Notes**
• The *Summary Table of Evidence* provides an efficient way of recording and presenting information obtained through assessment<br>• It has been designed to allow clinicians to view and compare information gathered from different elements of assessment, on one page, in order to identify 'at a glance' whether there is enough evidence to support a particular diagnosis, combination of diagnoses or not<br>• In addition, if diagnosis is not possible, the Summary Table of Evidence, may indicate which aspect of the assessment domains is lacking information. See pathway for assessment options<br>• The table can be completed by a diagnostician individually, although preferably it would be considered by more than one person<br>• To complete the form, use completed assessment tools, reports and proformas. Transfer the ratings given onto the Summary Table of Evidence using the key overleaf<br>• Once completed, this form should provide a summary of evidence relating to each of the diagnostic domains of DSM 5* diagnostic criteria<br><br>*American Psychiatric Association. (2013). *Diagnostic and statistical manual of mental disorders (DSM-5®)*. American Psychiatric Pub.
**DSM 5 requires the following for diagnosis of Developmental Co-ordination Disorder**
The following 4 criteria should be present:<br><br>A. Acquisition and execution of coordinated motor skills is substantially below what would be expected given the individual's chronological age and opportunity for skill learning and use; difficulties are manifested as clumsiness (e.g., dropping or bumping into objects) and as slowness and inaccuracy of performance of motor skills (e.g., catching an object, using scissor or cutlery, handwriting, riding a bike, or participating in sports)<br>B. The motor skills deficit in criterion A, significantly or persistently interferes with activities of daily living appropriate to the chronologic age (e.g., self-care and self-maintenance) and impacts academic/school productivity, prevocational and vocational activities, leisure, and play<br>C. The onset of symptoms is in the early developmental period<br>**D.** The motor skills deficits cannot be better explained by intellectual disability, intellectual developmental disorder, or visual impairment and are not attributable to a neurological condition affecting movement (e.g., cerebral palsy, muscular dystrophy, or a degenerative disorder)

Developmental Co-ordination Disorder

Child's name:
Date of birth:
Contact details/ CHI:
Date completed:
Completed by:

Key: Is this an area of challenge?
4 *None of the time*
3 *Some of the time*
2 *Most of the time*
1 *All of the time*

*American Psychiatric Association. (2013). *Diagnostic and statistical manual of mental disorders (DSM-5®)*. American Psychiatric Pub.

		PRIOR TO FIRST APPOINTMENT										FIRST APPOINTMENT				MEETS DIAGNOSTIC CRITERIA
**DSM Criteria**	**Diagnostic Items**	Early Developmental History		Physical examination				Reported observation by key informant from education setting				Narrative of Core Symptoms		Direct Observation by practitioner		
**A.**	Acquisition and execution of coordinated motor skills is substantially below what would be expected given the individual's chronological age and opportunity for skill learning and use; difficulties are manifested as clumsiness (e.g., dropping or bumping into objects) and as slowness and inaccuracy of performance of motor skills (e.g., catching an object, using scissor or cutlery, handwriting, riding a bike, or participating in sports)	Y	N	4	3	2	1	4	3	2	1	Y	N	Y	N	
**B.**	The motor skills deficit in criterion A, significantly or persistently interferes with activities of daily living appropriate to the chronologic age (e.g., self-care and self-maintenance) and impacts academic/school productivity, prevocational and vocational activities, leisure, and play	Y	N	4	3	2	1	4	3	2	1	Y	N	Y	N	
**C.**	The onset of symptoms is in the early developmental period	Y	N	4	3	2	1	4	3	2	1	Y	N	Y	N	
**D.**	The motor skills deficits cannot be better explained by intellectual disability, intellectual developmental disorder, or visual impairment and are not attributable to a neurological condition affecting movement (e.g., cerebral palsy, muscular dystrophy, or a degenerative disorder)	Y	N	4	3	2	1	4	3	2	1	Y	N	Y	N	

Outcome

◯ **Meets diagnostic criteria** ◯ **Does not meet diagnostic criteria** ◯ **Diagnosis unclear;** undertake further assessment or review in ___ months

Summary Table of Evidence: Fetal Alcohol Spectrum Disorder

Summary Table of Evidence: Fetal Alcohol Spectrum Disorder*

Guidance Notes

- The *Summary Table of Evidence* provides an efficient way of recording and presenting information obtained through assessment
- It has been designed to allow clinicians to view and compare information gathered from different elements of assessment, on one page, in order to identify 'at a glance' whether there is enough evidence to support a particular diagnosis, combination of diagnoses or not
- In addition, if diagnosis is not possible, the Summary Table of Evidence, may indicate which aspect of the assessment domains is lacking information. See pathway for assessment options
- The table can be completed by a practitioner individually, although preferably it would be considered by more than one person
- To complete the form, use completed assessment tools, reports and proformas. Transfer the ratings given onto the Summary Table of Evidence using the key overleaf
- Once completed, this form should provide a summary of evidence relating to each of the diagnostic criteria
- The SIGN 156 Guideline group developed an algorithm for diagnosis linked to ICD criteria https://d3pw27xtndcm0o.cloudfront.net/Uploads/q/g/u/ginpodiagnosticalgorithmforfasd_496096.pdf
- Within this algorithm the following are possible descriptors:
    - **FASD without sentinel facial features**
    - **FASD with sentinel facial features**
    - **At Risk of FASD**
- DSM 5 uses the following terminology:
    - Other Specified Neurodevelopmental Disorder (315.8) alongside a qualifier e.g. "Other Specified Neurodevelopmental Disorder (associated with prenatal alcohol exposure) or ND-PAE.

SIGN 156 requires the following for diagnosis of FASD (with or without sentinel facial features)

The following criteria should be fulfilled :

A. Prenatal alcohol exposure confirmed or unknown but suspected

B. There are 3 sentinel facial features which may be present and indicative of this diagnosis. These are:
- Palpebral fissure length (PFL) < 3rd centile
- Thin upper lip (rank 4 or 5 of the University of Washington Lip-Philtrum Guide)
- Smooth philtrum (rank 4 or 5 of the University of Washington Lip-Philtrum Guide)
- Practitioners should note whether a) less than 3 are present b) all 3 are present.

Presence supports diagnosis of **FASD with sentinel features**. Absence can still lead to the more prevalent/ or more common diagnosis of **FASD without sentinel features**

C. Evidence of pervasive and long-standing brain dysfunction, which is defined by severe impairment (a global score or a major subdomain score on a standardised neurodevelopmental measure that is ≥2 SDs below the mean, with appropriate allowance for test error) in three of more of the following areas of assessment:
- motor skills
- neuroanatomy/neurophysiology
- cognition
- language**
- academic achievement
- memory
- attention
- executive function, including impulse control and hyperactivity
- affect regulation
- adaptive behaviour, social skills or social communication

**NB: Impairment is also present when there is a large discrepancy between receptive and expressive language composite scores and the lower of the two discrepant scores is at least one standard deviation below the mean.

D. For infants and children under 6 years, pre-natal alcohol exposure together with microcephaly is a clear diagnostic indicator. If microcephaly is not present but other factors are, they should be considered **'At risk' for FASD**

*NB: Current guidance is that the DSM 5 terminology is only for use for research purposes. In practice in Scotland SIGN 156 recommends use of the DSM category of ***Other Specified Neurodevelopmental Disorder*** and the use of the descriptor ***associated with Alcohol Exposure***

Fetal Alcohol Spectrum Disorder

Child's name:
Date of birth:
Contact details/ CHI:
Date completed:
Completed by:

Key: Is this an area of challenge?
4 *None of the time*
3 *Some of the time*
2 *Most of the time*
1 *All of the time*

Indicators for Diagnosis / Diagnostic Items	PRIOR TO FIRST APPOINTMENT												FIRST APPOINTMENT				MEETS DIAGNOSTIC CRITERIA
	Early Developmental, Family and Social History		Reported observation by key informant and /or individual				Reported observation by key informant from education setting				Physical Examination		Narrative of Core Symptoms		Direct Observation by practitioner		
**A.** Prenatal alcohol exposure confirmed or known	Y	N											Y	N	Y	N	
**B.** Presence of sentinel features											Y	N			Y	N	
1. Short palpebral fissure length (PFL)											Y	N			Y	N	
2. Thin upper lip											Y	N			Y	N	
3. Smooth Philtrum											Y	N			Y	N	
**C.** Evidence of pervasive and long-standing brain dysfunction, which is defined by severe impairment (a global score or a major subdomain score on a standardised neurodevelopmental measure that is ≥2 SDs below the mean, with appropriate allowance for test error) in three of more of the following areas of assessment (see SIGN 156 for full detail and guidance):	Y	N	4	3	2	1	4	3	2	1			Y	N	Y	N	
• motor skills	Y	N	4	3	2	1	4	3	2	1	Y	N	Y	N	Y	N	
• neuroanatomy/neurophysiology	Y	N									Y	N	Y	N	Y	N	
• cognition	Y	N	4	3	2	1	4	3	2	1			Y	N	Y	N	
• language	Y	N	4	3	2	1	4	3	2	1			Y	N	Y	N	
• academic achievement	Y	N	4	3	2	1	4	3	2	1			Y	N	Y	N	
• memory	Y	N	4	3	2	1	4	3	2	1			Y	N	Y	N	
• attention	Y	N	4	3	2	1	4	3	2	1			Y	N	Y	N	
• executive function, including impulse control and hyperactivity	Y	N	4	3	2	1	4	3	2	1			Y	N	Y	N	
• affect regulation	Y	N	4	3	2	1	4	3	2	1			Y	N	Y	N	
• adaptive behaviour, social skills or social communication	Y	N	4	3	2	1	4	3	2	1			Y	N	Y	N	
**D.** Microcephaly is present	Y	N									Y	N					
**E.** History of Epilepsy	Y	N									Y	N					
**F.** There is clear evidence that the symptoms interfere with, or reduce the quality of, social, school, or work functioning.	Y	N	4	3	2	1	4	3	2	1			Y	N	Y	N	
**G.** Has consideration been given to whether the symptoms co-occur with or have possible contribution of genetic, physical, neurodevelopmental or mental disorders or adverse childhood experiences	Y	N											Y	N	Y	N	

Supplementary File S4: views from people with lived experience

Theme 1: Keep families of individuals being assessed informed at every stage of the process

Families have told us that they can easily become anxious and concerned if they think ‘nothing is happening’ and this will be especially true for parents/ carers if their child is presenting ‘clear difficulties’ and causing real family concern. Much of the information gathering will be going on behind the scenes and parents are likely be quite unaware of the depth, diversity and complexities of this input. Make sure families understand this, through providing information in range of ways. Remember that being told once may not be enough for people to hold and make sense of the information. It is important to check that the message intended is the one understood by families. Service providers should endeavor to provide timely information at the level and in the amount suitable for all families.

Theme 2: Keep individuals being assessed informed about the process in a developmentally relevant way

Service providers and the adults around the child, should endeavor to provide information at the level and in the amount suitable for the individual child or young person. Those at an earlier developmental stage, may need information at a simple level and linked to the here and now. Some children and young people may not need to know they are being ‘assessed’ or may not understand what this means. They may benefit from visual supports in preparation for an appointment on the days leading up to it. The clinic team may be able to provide these, for example pictures of the place they are going or the people they might see. They may need to know:

- they will go in the car/ on the bus to a place
- they will see some new/ familiar people to play/ look at books/ talk
- that their parents will talk to the new people too
- that they can take a snack/drink/ favourite toy/ activity
- that their parents will stay with them/ or not if they have to attend without them in the room
- when it’s finished they will go home

Think about the words or visuals you use, for example if you say we are going to the hospital or a doctor, they may have other associations with these words. There may be alternative words you can use that describe what the child will experience. For those at a later developmental stage it may be appropriate to do the same as above and to provide information or answer queries on a need to know basis – ie what is relevant and important to them rather than all of the things the adults know or think. Individualised Social Stories may be helpful for some, to help them to understand what to expect, when and with whom. Some individuals may be aware that they are undergoing assessment and understand why. Some may not. Some

individuals may be aware that they experience challenges or that they do some things differently from many of their peers. Some may not. Some children and young people will be ok with going along with their parents without questioning why, others may be seeking a diagnosis and understanding of their own differences and others may not wish to take part in the assessment process.

Reflect on the nature and type of information shared with families and individuals. Service providers, together with those accessing services can consider:

a) How clear and accessible is the information about the diagnostic assessment process?
b) How predictable is the assessment process? Do things happen at the time and in the way families and individualsexpect? Do different professionals give consistent information?
c) How can the process be made more positive/ desirable?

Theme 3: Make the request for assessment and support process clear to families, especially when the decision is made not to proceed with diagnostic assessment

It is important that those seeking assessment understand how to access this support at different stages of concerns being identified. When concerns are raised (particularly by parents) and the criteria for assessment is not met, there may be feelings of confusion and upset. Being rejected by people who have never seen (and are not going to see) your child can feel quite dismissive. Reflect on the request for assessment process. Questions might include:

a) Are individuals/ families clear about how to request assessment and are they supported to do so? Do they know why the assessment is proceeding or not?
b) Have they been made aware that a further request can be made if circumstances/ presentations change or they disagree with the decision? and
c) Is information provided and support in place to address concerns raised and challenges experienced by the individual and family? – what is this and is it helpful?

Theme 4: **Help set expectations about waiting times and provide a point of contact**

For different individuals in different areas, there can be a wide range of timescales in accessing different steps in the pathway. Explain the process of assessment to families, along with plans to provide immediate support. When there is going to be a period of time to wait for particular appointments, let families know how long that is likely to be (even if this is an approximate time) and who to contact/ how to contact them if they have not heard after a period of time.

Reflect on how well shared expectations are communicated with and understood by families. For example

a) do families feel informed?
b) are families signposted to seek a child or young person's planning meeting and other forms of relevant support?
c) do professionals have access to relevant, accurate information about waiting times and sources of support?

Theme 5: At assessment appointments

The experience on the day of an appointment is also important to individuals and families who may have had to make many plans and adjustments to get there.

- If possible, set your environment up for success. Remove things from reach that should not be touched by the child or young person and create a calm, safe and clutter free space with resources that provide opportunity for social communication and sensory regulation.
- Avoid unnecessary waiting – start and finish at the time you have said and keep appointments to a duration that is manageable at this age and stage.
- If possible, ensure the child is not present or is well occupied during parent interviews.
- If possible, avoid unnecessary duplication by ensuring professionals have accessed and read relevant previous reports.
- Start by clarifying the family and individual's aspirations and expectations of the appointment, what will happen, the duration of the appointment. Give them an opportunity to ask questions and raise issues at the start and ensure they know about further opportunities to ask questions following this appointment.
- Actively listen to the individual and family.

Reflect on feedback from those who attend appointments and ask them about a) what worked well/ could be better - in advance of the appointment, at the appointment and after it b) the environment in the 'clinic' setting and c) the communication of the professionals they meet

Theme 6: no supports should be diagnosis dependent

Families might falsely believe that they can only get the support they need following a diagnosis. Reinforce the message that no support is diagnosis dependent, with families/ individuals and consider whether they are sufficiently supported with practical holistic help whilst waiting for assessment. This is particularly necessary if the waiting list is lengthy. Parents can almost be in a holding pattern during this period and the assessment day can loom large and become a destination as opposed to an information day.

Before, during and after diagnosis, individualised planning is essential, for example although parent and family supports may be offered – do these actually meet the needs of this family or of children with neurodevelopmental needs. Some programmes or interventions may leave families feeling like a failure because the strategies are not suitable for their child's presentation, or they may be at a fairly universal level when the individual and family need a much more targeted approach.

Reflect on support provided according to need within your service. You could consider a) does your service limit access to supports before diagnosis and if so how can this be addressed? b) are supports offered before, during and after diagnosis developmentally relevant and individualised to meet particular needs or is there a bit of a 'one size fits all' approach? c) how well are health, education and other services working together to meet the range of needs that arise locally and d) are families potentially wasting time on attending programmes that are not right for them or finding out information that is not relevant?

Theme 7: Quality and timing of information provided is more important than quantity

On the day a diagnosis is shared, parents or children and young people are unlikely to absorb great tranches of information. Each individual will take in and remember different bits of information from what they hear. It is important to check what they have understood and to link them to ongoing sources of support in their local area and to the professionals they will link with.

As well as receiving information, individuals may need the opportunity to ask questions and discuss their understanding of how this relates to them with people with the relevant expertise and knowledge.

Timing of such opportunities to receive and discuss information is important and totally dependent on the individuals involved. Different information is usually required over time as the child or young person grows through new stages.

A range of accessible material is best (written information, videos or websites) and our mantra would be quality and relevance, not quantity, of information. Reflect on:

a) what information is shared?
b) how is information shared with families?
c) is this accessible?
d) are there opportunities both on the day of diagnosis and after, for the individual and family to have conversations with health and education professionals with suitable experience and knowledge about both the diagnosis and its implications and the range of financial, health, educational or other supports?

Theme 8: Parents don't know what they don't know

As well as really listening to parents, professionals need to provide guidance and orientation to families to help them identify what is most likely to be important and essential for them to know at different points in time. Parents tell us they can waste years trying to source information only to find out it wasn't that relevant. Avoid putting pressure on families to come up with ideas and questions or to articulate what they need in terms of support without providing some frames of reference. It might help to signpost families to local supports as necessary.
